# Mac-2 binding protein glycosylation isomer contributes to identify advanced Fontan-associated liver disease: A retrospective cohort study

**DOI:** 10.1097/MD.0000000000049224

**Published:** 2026-06-05

**Authors:** Kensuke Kitsugi, Yoshisuke Hosoda, Go Murohisa, Yashiro Yoshizawa, Masaharu Kimata, Yosuke Kobayashi, Shuhei Unno, Yosuke Yamada, Toshihiro Takayanagi, Kazuhito Kawata

**Affiliations:** a Department of Gastroenterology, Seirei Hamamatsu General Hospital, Shizuoka, Japan; b Department of Internal Medicine II, Hamamatsu University School of Medicine, Shizuoka, Japan.

**Keywords:** brain natriuretic peptide, central venous pressure, Fontan procedure, liver cirrhosis

## Abstract

The usefulness of several noninvasive assessments for evaluating liver fibrosis has been reported in Fontan-associated liver disease (FALD). However, no studies have reported on the usefulness of Mac-2 binding protein glycosylation isomer (M2BPGi) in FALD. Here, we investigated the usefulness of M2BPGi in identifying advanced FALD. This retrospective study included patients diagnosed with FALD. Based on the clinical signs of portal hypertension, we defined advanced FALD as a varices, ascites, splenomegaly, and thrombocytopenia (VAST) score ≥ 2, and we compared the cardiac parameters, liver parameters, and serological fibrosis markers, including M2BPGi. The Mann-Whitney U and Fisher exact test were used to compare variables. Spearman rank correlation coefficients (r) were calculated to evaluate correlations. The accuracy of outcome prediction was evaluated using the area under the receiver operating characteristic curve (AUROC). A total of 43 patients with FALD were enrolled. There were 22 cases (51%) diagnosed with advanced FALD. In the serological fibrosis markers, M2BPGi levels were significantly higher in cases with advanced FALD (median 0.65 vs 0.38 cutoff index [C.O.I], *P* = .003), similar to the fibrosis-4 (FIB-4) index and aspartate aminotransferase (AST) to platelet ratio index (APRI). Receiver operating characteristic analysis revealed that M2BPGi demonstrated moderate ability to predict the cases with advanced FALD (AUROC, 0.767), and the cutoff value was 0.64 C.O.I. Moreover, M2BPGi demonstrated a significant correlation with AST (*r* = 0.321, *P* = .036), albumin (*r* = −0.562, *P* < .001), FIB-4 index (*r* = 0.492, *P* = .001), APRI (*r* = 0.399, *P* = .008), and cardiac parameters such as central venous pressure (*r* = 0.413, *P* = .006) and brain natriuretic peptide (*r* = 0.431, *P* = .004). Esophagogastric varices (31% vs 3%, *P* = .024) and ascites (69% vs 27%, *P* = .016) were more prevalent in the higher M2BPGi group. Furthermore, there was a significant trend in increasing M2BPGi with worsening the VAST scores (*P* = .021). This study suggests that M2BPGi may be useful for identifying advanced FALD.

## 1. Introduction

The Fontan procedure is a palliative operation for patients with congenital heart disease with a functional single ventricle.^[1]^ Improvements in surgical techniques and medical management have improved long-term outcomes after the Fontan procedure. However, complications resulting from Fontan circulation, which is caused by anastomosis between the vena cava or right atrium and the pulmonary arteries, have become an issue in patients with post-Fontan procedure.

High central venous pressure (CVP) and reduced cardiac output due to poor venous return caused by Fontan circulation can lead to congestion and ischemia in the liver, developing Fontan-associated liver disease (FALD).^[2]^ A previous study using liver biopsy revealed that fibrosis was observed in all cases of FALD, with advanced fibrosis in 36% of cases.^[3]^ Moreover, the incidence of hepatocellular carcinoma (HCC) in patients with FALD is estimated to be 1.2% in a nationwide survey in Japan, which worsens the prognosis of FALD.^[4]^ As in chronic liver disease in adults, the progression of liver fibrosis is considered an important risk factor for HCC in FALD.^[5]^

Liver biopsy is the gold standard for the diagnosis of liver fibrosis. However, congestive hepatopathy and the presence of antithrombotic agents, often used in patients with FALD, increase the risk of bleeding.^[6]^ In recent years, the usefulness of elastography and serological fibrosis markers has been reported as noninvasive assessments for the diagnosis of liver fibrosis. However, elastography is affected by liver congestion and may overestimate liver stiffness.^[7,8]^ There is no consensus on serological fibrosis markers, but some reports have demonstrated that the Fibrosis-4 (FIB-4) index and aspartate aminotransferase (AST) to platelet ratio index (APRI) are useful for diagnosing FALD in patients with advanced fibrosis.^[9,10]^

Mac-2 binding protein glycosylation isomer (M2BPGi) is a new serological fibrosis marker that is attracting attention for its usefulness in diagnosing the progression of liver fibrosis and predicting the risk of HCC. M2BPGi is a glycoprotein produced by hepatic stellate cells (HSCs) that functions as a messenger between HSCs and Kupffer cells to promote fibrogenesis.^[11]^ M2BPGi has been reported to be useful for staging liver fibrosis in chronic liver diseases, particularly chronic hepatitis C.^[12]^ However, the cutoff value varies depending on the underlying liver disease. For example, the cutoff value for M2BPGi in identifying severe fibrosis is reported to be 4.0 cutoff index (C.O.I) for patients with hepatitis C virus (HCV), while it is reported to be 1.2 C.O.I for patients with metabolic dysfunction-associated steatotic liver disease (MASLD).^[12,13]^ To the best of our knowledge, there have been no reports on the usefulness of M2BPGi in FALD, and the cutoff value for identifying advanced cases is unknown. In this study, we investigated the usefulness of M2BPGi in identifying advanced FALD.

## 2. Methods

### 2.1. Patients

This was a retrospective observational study. The study population consisted of patients with FALD who were assessed for M2BPGi at Seirei Hamamatsu General Hospital between April 2017 and March 2025. FALD was diagnosed based on liver morphological abnormalities on imaging tests or liver dysfunction on laboratory tests, as previously reported.^[14]^ Specifically, the patients were diagnosed with FALD if any of the following conditions were met: aspartate aminotransferase (AST) or alanine aminotransferase (ALT) ≥ 30 U/L, gamma-glutamyl transferase (GGT) ≥ 50 U/L, total bilirubin ≥ 1.2 mg/dL, hepatomegaly or hepatic atrophy, enlargement of the caudate lobe, and liver surface nodularity. The exclusion criteria were as follows: coexistence of other liver diseases, such as fatty liver or viral hepatitis, not referred to hepatologists, cases with missing data. The remaining 43 patients were enrolled in this study. This study was approved by the Ethics Committee of the Hamamatsu University Hospital (Ethics Approval Number: 4635). Each patient was offered the opportunity to decline participation in the study through an opt-out option.

### 2.2. Evaluations

The clinical and demographic characteristics of the patients and laboratory data were collected from medical records. We investigated patient characteristics, including age at the time of evaluation and the Fontan procedure, sex, duration of Fontan circulation, body mass index, underlying congenital heart disease, and the presence of antithrombotic agents. Cardiologic parameters such as CVP, ejection fraction (EF), O2 saturation, and brain natriuretic peptide (BNP) were obtained from physical examinations, blood tests, and cardiac catheterization performed within 1 year before and after the assessment of liver parameters. We also investigated the type of Fontan procedure (atriopulmonary connection [APC] or total cavopulmonary connection [TCPC]). Based on the European Reference Network on Rare Liver Diseases (EASL-ERN) position paper,^[15]^ advanced FALD was diagnosed based on the clinical signs of portal hypertension. Specifically, a varices, ascites, splenomegaly, and thrombocytopenia (VAST) score ≥ 2 was defined as advanced FALD. The VAST score was calculated as the sum of clinical findings of esophagogastric varices, ascites, splenomegaly, and thrombocytopenia (platelet count < 15 × 10^4^/μL).^[16]^ We evaluated the following complications: asplenia, polysplenia, protein-losing enteropathy, splenomegaly, esophagogastric varices, ascites, and HCC. Esophagogastric varices and ascites were diagnosed using imaging modalities. Splenomegaly was diagnosed by imaging if the maximum diameter was >13 cm. Liver function tests included the evaluation of total bilirubin, AST, ALT, alkaline phosphatase (ALP), GGT, albumin, and platelet count. Regarding serological markers of liver fibrosis, we also evaluated the FIB-4 index and APRI, along with M2BPGi. M2BPGi levels were measured using the HISCL-5000 immunoanalyzer (Sysmex, Hyogo, Japan) and expressed as C.O.I. FIB-4 index and APRI were calculated by the following formulas:^[17,18]^

FIB-4 index = age (years) × AST level (U/L)/platelet count (10^3^/μL) × ALT level (U/L)^1/2^

APRI = [{AST (U/L)/upper normal limit of AST (U/L)}/platelet count (10^3^/μL)].

### 2.3. Statistical analyses

Data on patient characteristics are presented as numbers for categorical data and medians and interquartile ranges for continuous variables. Nonparametric analyses were performed without excluding the outliers. The Mann–Whitney U test was used to compare continuous variables. Categorical variables were compared using Fisher exact test. Spearman rank correlation coefficients (*r*) were calculated to evaluate correlations. The accuracy of the outcome prediction was evaluated by the area under the receiver operating characteristic curve (AUROC), and the best cutoff value was calculated using the Youden Index. Receiver operating characteristic (ROC) analyses were performed without adjusting for covariates. DeLong test was conducted to compare the predictive powers. A trend test was performed using the Jonckheere-Terpstra test. All analyses were performed using EZR, a modified version of the R commander designed to add statistical functions frequently used in biostatistics.^[19]^ Statistical significance was set at *P* < .05.

## 3. Results

### 3.1. Patient characteristics

The baseline patient characteristics are summarized in Table 1. The median age at evaluation was 18.0 years, and at the Fontan procedure was 2.0 years. Males comprised 26 cases (61%), and females comprised 17 cases (39%). The median time since the Fontan procedure was 15.0 years. The median body mass index was 19.6 kg/m^2^, with 2 cases above 25.0 kg/m^2^. The most common etiology of underlying congenital heart disease was double-outlet right ventricle with 14 cases (33%), followed by pulmonary atresia with 11 cases (25%), single cardiac ventricle with 9 cases (21%), and tricuspid valve insufficiency with 9 cases (21%). Five cases (12%) had asplenia, one (2%) had polysplenia, 4 cases (9%) had protein-losing enteropathy. Splenomegaly, esophagogastric varices, and ascites were present in 22 (61%), 6 (14%), and 17 (40%) cases, respectively. None of the patients had HCC. The Fontan procedure was APC in only 3 cases (7%) and TCPC in most cases (40 cases, 93%). In the cardiac parameters, the median CVP was 13 mm Hg, EF was 53.7%, O2 saturation was 93%, and BNP was 11.8 pg/mL. Most cases (38 cases, 88%) received antithrombotic drugs. In the laboratory data, the median values of total bilirubin, AST, ALT, and albumin were 1.1 mg/dL, 25 U/L, 23 U/L, and 4.6 g/dL respectively, and most cases were within the normal range. In contrast, the median values of ALP and GGT were 101 U/L and 66 U/L, and abnormal values were observed in 19 (44%) and 31 (72%) cases, respectively. The median platelet count was 16.7 × 10^4^/μL, with 18 cases (42%) below 15.0 × 10^4^/μL. In the serological fibrosis markers, the median values of M2BPGi, FIB-4 index, and APRI were 0.43 C.O.I, 0.66, and 0.38, respectively. Twenty-two cases (51%) had a VAST score ≥ 2 and were diagnosed with advanced FALD.

**Table 1:** Baseline demographic and clinical characteristics in patients with FALD (n = 43).

Variables	Results
Age at evaluation [years]	18.0 (17.0–27.0)
Age at the Fontan procedure [years]	2.0 (1.5–7.0)
Sex, n (%)	
Male	26 (61)
Female	17 (39)
Duration of Fontan circulation [years]	15.0 (13.0–17.5)
Body mass index [kg/m^2^]	19.6 (18.0–22.3)
Cardiac disease, n (%)	
Double-outlet right ventricle	14 (33)
Pulmonary atresia	11 (25)
Single cardiac ventricle	9 (21)
Tricuspid valve insufficiency	9 (21)
Complications, n (%)	
Asplenia	5 (12)
Polysplenia	1 (2)
Protein losing enteropathy	4 (9)
Splenomegaly	22 (61)
Esophagogastric varices	6 (14)
Ascites	17 (40)
Hepatocellular carcinoma	0(0)
Fontan type, n (%)	
Total cavopulmonary connection	40 (93)
Atriopulmonary connection	3 (7)
Cardiac parameters	
Central venous pressure (mm Hg)	13 (8–22)
Ejection fraction (%)	53.7 (48.5–60.4)
O_2_ saturation (%)	93 (92–95)
Brain natriuretic peptide (pg/mL)	11.8 (6.0–28.0)
Antithrombotic agents, n (%)	
Antiplatelet agents	17 (40)
Anticoagulant agents (warfarin or direct oral anticoagulants)	11 (25)
Combination of antiplatelet and anticoagulant agents	10 (23)
No antithrombotic agent	5 (12)
Laboratory data	
Total bilirubin [mg/dL]	1.1 (0.8–1.9)
Aspartate aminotransferase [U/L]	25 (21–29)
Alanine aminotransferase [U/L]	23 (19–32)
Alkaline phosphatase [U/L]	101 (76–144)
Gamma-glutamyl transferase [U/L]	66 (44–92)
Albumin [g/dL]	4.6 (4.3–4.9)
Platelet count [x10^4^/μL]	16.7 (12.9–19.3)
Serological fibrosis markers	
M2BPGi [C.O.I]	0.43 (0.32–0.68)
FIB-4 index	0.66 (0.45–0.88)
APRI	0.38 (0.30–0.51)
VAST score, n (%)	
0	11 (26)
1	10 (23)
2	15 (35)
3	6 (14)
4	1 (2)

Data are presented as numbers for categorical data, and medians with interquartile ranges for continuous variables.

APRI = aspartate aminotransferase to platelet ratio index, C.O.I = cutoff index, FIB-4 = fibrosis-4, M2BPGi = Mac-2 binding protein glycosylation isomer, VAST = varices, ascites, splenomegaly, thrombocytopenia.

### 3.2. Comparison between cases with advanced and non-advanced FALD

The comparison of patient characteristics between advanced and non-advanced FALD is summarized in Table 2. There was no significant difference in the age at evaluation, whereas the age at the Fontan procedure was significantly higher in cases with advanced FALD (median 4.5 vs 2.0 years, *P* = .017). There were no significant differences in the time from the Fontan procedure or the etiology of the underlying congenital heart disease. Splenomegaly (*P* < .001), esophagogastric varices (*P* = .048), and ascites (*P* < .001), all of which are included in the VAST score, were significantly more common in cases with advanced FALD. There were no significant differences between the 2 groups in terms of the type of Fontan procedure, cardiac parameters, or use of antithrombotic agents. In the laboratory data, only platelet count was significantly lower in cases with advanced FALD (median 13.3 × 10^4^ vs 18.6 × 10^4^/μL, *P* = .002). Regarding the serological fibrosis markers, M2BPGi (median 0.65 vs 0.38 C.O.I, *P* = .003), FIB-4 index (median 0.79 vs 0.46, *P* < .001), and APRI (median 0.48 vs 0.32, *P* = .001) were significantly higher in cases with advanced FALD.

**Table 2:** Comparison of patient characteristics between advanced and non-advanced FALD.

Variables	Advanced FALD (n = 22)	Non-advanced FALD (n = 21)	*P*-value
Age at evaluation [years]	22.0 (17.0–29.3)	17.0 (17.0–18.0)	.081
Age at the Fontan procedure [years]	4.5 (2.0–10.8)	2.0 (1.0–3.0)	.017
Sex, n (%)			1.000
Male	13 (59)	13 (62)	
Female	9 (41)	8 (38)	
Duration of Fontan circulation [years]	15.0 (11.3–18.8)	16.0 (14.0–17.0)	.575
Body mass index [kg/m^2^]	19.8 (19.0–22.1)	19.2 (17.5–22.1)	.512
Cardiac disease, n (%)			.086
Double-outlet right ventricle	4 (18)	9 (43)	
Pulmonary atresia	8 (36)	3 (14)	
Single cardiac ventricle	7 (32)	3 (14)	
Tricuspid valve insufficiency	3 (14)	6 (29)	
Complications, n (%)			
Asplenia	2 (9)	3 (14)	.664
Polysplenia	1 (5)	0 (0)	1.000
Protein losing enteropathy	2 (9)	2 (10)	1.000
Splenomegaly	18 (95)	4 (24)	<.001
Esophagogastric varices	23 (5)	0 (0)	.048
Ascites	15 (68)	2 (10)	<.001
Fontan type, n (%)			1.000
Total cavopulmonary connection	20 (91)	20 (95)	
Atriopulmonary connection	2 (9)	1 (5)	
Cardiac parameters			
Central venous pressure (mm Hg)	13.0 (11.0–16.0)	12.0 (11.0–14.0)	.347
Ejection fraction (%)	55.2 (49.0–59.7)	52.0 (47.9–60.1)	.706
O2 saturation (%)	92.3 (91.1–94.9)	94.0 (92.0–95.0)	.385
Brain natriuretic peptide (pg/mL)	20.1 (6.1–54.4)	9.0 (6.2–13.7)	.101
Antithrombotic agents, n (%)			.808
Antiplatelet agents	8 (36)	9 (43)	
Anticoagulant agents (warfarin or direct oral anticoagulants)	7 (32)	4 (19)	
Combination of antiplatelet and anticoagulant agents	5 (23)	5 (24)	
No antithrombotic agent	2 (9)	3 (14)	
Laboratory data			
Total bilirubin [mg/dL]	1.1 (0.9–1.9)	0.9 (0.8–1.3)	.256
Aspartate aminotransferase [U/L]	27 (21–32)	24 (21–26)	.184
Alanine aminotransferase [U/L]	23 (19–30)	23 (18–32)	.874
Alkaline phosphatase [U/L]	100 (70–155)	101 (82–120)	.884
Gamma-glutamyl transferase [U/L]	69 (46–110)	62 (44–74)	.481
Albumin [g/dL]	4.5 (4.2–4.9)	4.7 (4.5–4.9)	.157
Platelet count [x10^4^/μL]	13.3 (11.8–17.2)	18.6 (16.7–20.3)	.002
Serological fibrosis markers			
M2BPGi [C.O.I]	0.65 (0.39–0.87)	0.38 (0.30–0.47)	.003
FIB-4 index	0.79 (0.65–1.16)	0.46 (0.40–0.66)	<.001
APRI	0.48 (0.38–0.61)	0.32 (0.25–0.38)	.001

Data are presented as numbers for categorical data, and medians with interquartile ranges for continuous variables.

APRI = aspartate aminotransferase to platelet ratio index, C.O.I = cutoff index, FALD = Fontan-associated liver disease, FIB-4 = fibrosis-4, M2BPGi = Mac-2 binding protein glycosylation isomer.

### 3.3. Evaluation of the utility of M2BPGi in predicting advanced FALD

**Figure 1.**
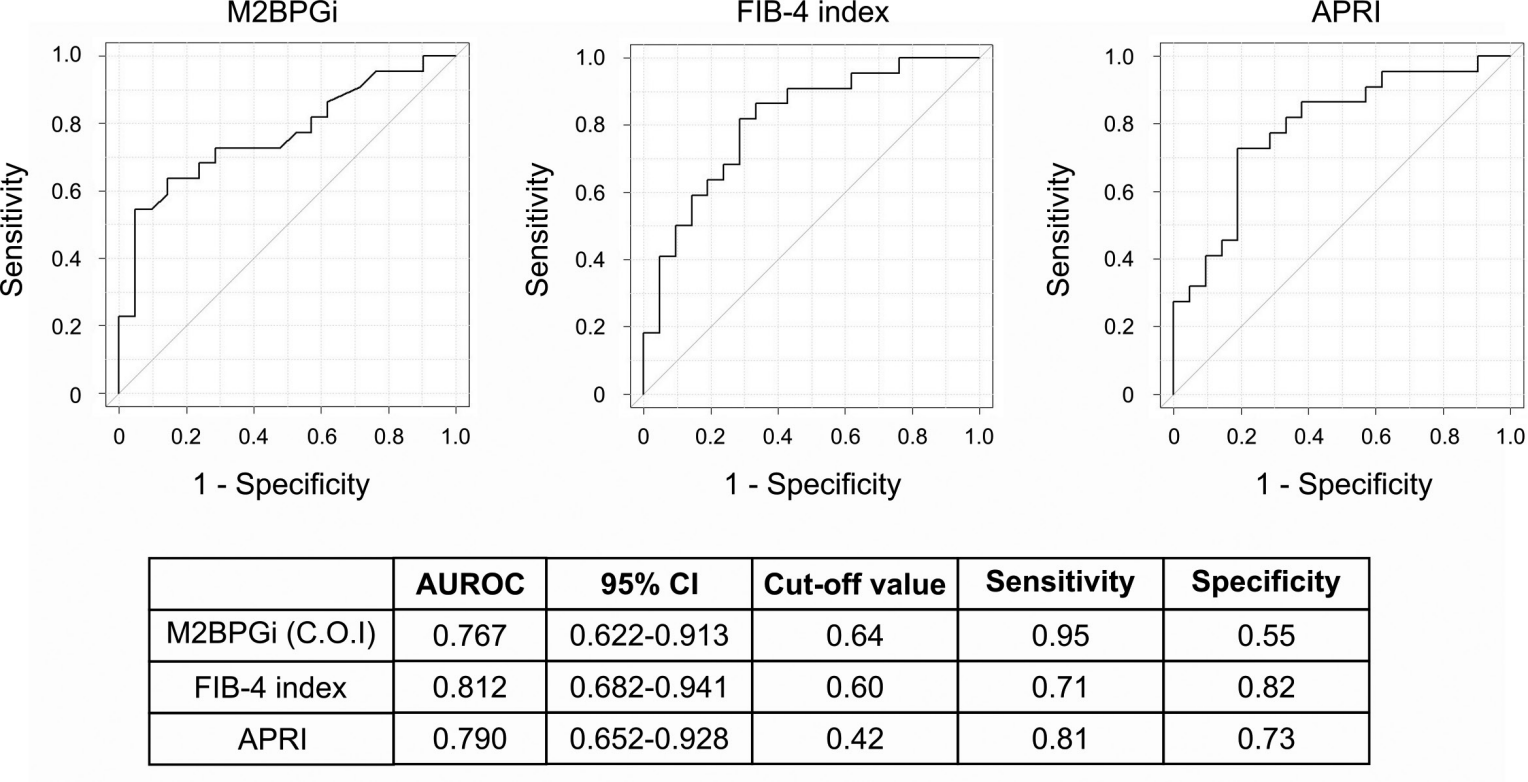
ROC analysis of M2BPGi, FIB-4 index, and APRI to predict the cases with advanced FALD. FALD, Fontan-associated liver disease; ROC, receiver operating characteristic; M2BPGi, Mac-2 binding protein glycosylation isomer; C.O.I, cutoff index; FIB-4, fibrosis-4; APRI, aspartate aminotransferase to platelet ratio index; AUROC, area under the receiver operating characteristic; CI, confidence interval.

Next, we performed ROC analyses to evaluate the diagnostic performance of M2BPGi in cases with advanced FALD. As shown in Figure 1, M2BPGi demonstrated moderate ability to predict advanced FALD (AUROC, 0.767), and the cutoff value was 0.64 C.O.I. FIB-4 index and APRI also demonstrated moderate ability to predict advanced FALD cases (AUROC: FIB-4 index, 0.812; APRI, 0.790). DeLong test demonstrated that M2BPGi and these fibrosis markers were comparable in predicting advanced FALD (M2BPGi vs FIB-4 index, *P* = .60; M2BPGi vs APRI, *P* = .80).

### 3.4. Evaluation of the correlation of M2BPGi with other clinical parameters

Next, we evaluated the relationship between M2BPGi levels and other clinical parameters. In categorical variables, the higher M2BPGi group (M2BPGi ≥ 0.64 C.O.I) had significantly lower rates of tricuspid valve insufficiency in underlying congenital heart disease (0% vs 30%, *P* = .039). Moreover, esophagogastric varices (31% vs 3%, *P* = .024) and ascites (69% vs 27%, *P* = .016) were significantly more prevalent in the higher M2BPGi group (Table 3). Among the other continuous variables, M2BPGi demonstrated a significant correlation with age at the Fontan procedure (*r* = 0.558, *P* < .001), CVP (*r* = 0.413, *P* = .006), BNP (*r* = 0.431, *P* = .004), AST (*r* = 0.321, *P* = .036), and albumin (*r* = −0.562, *P* < .001) (Table 4). In the serological fibrosis markers, M2BPGi was significantly correlated with FIB-4 index (*r* = 0.492, *P* = .001) and APRI (*r* = 0.399, *P* = .008) (Table 4). In the correlation between serological fibrosis markers and cardiac parameters, M2BPGi significantly correlated with both CVP and BNP, whereas the FIB-4 index demonstrated a significant correlation only with BNP (CVP, *r* = 0.291, *P* = .058; BNP, *r* = 0.546, *P* < .001), and APRI demonstrated no significant correlation with either CVP or BNP (CVP, *r* = 0.299, *P* = .051; BNP, *r* = 0.240, *P* = .12).

**Table 3:** Comparison of categorical variables compared to the value of M2BPGi.

Variables	Higher M2BPGi group (n = 13)	Lower M2BPGi group (n = 30)	*P*-value
Sex, n (%)			.310
Male	6 (46)	20 (67)	
Female	7 (54)	10 (33)	
Cardiac disease, n (%)			.014
Double-outlet right ventricle	2 (15)	11 (36)	
Pulmonary atresia	6 (46)	5 (17)	
Single cardiac ventricle	5 (39)	5 (17)	
Tricuspid valve insufficiency	0 (0)	9 (30)	
Complications, n (%)			
Asplenia	2 (15)	3 (10)	.630
Polysplenia	0 (0)	1 (3)	1.000
Protein losing enteropathy	2 (15)	2 (7)	.572
Splenomegaly	8 (80)	14 (54)	0.255
Esophagogastric varices	4 (31)	1 (3)	.024
Ascites	9 (69)	8 (27)	.016
Fontan type, n (%)			1.000
Total cavopulmonary connection	12 (92)	28 (93)	
Atriopulmonary connection	1 (8)	2 (7)	
Antithrombotic agents, n (%)			1.000
Antiplatelet agents	5 (39)	12 (40)	
Anticoagulant agents (warfarin or direct oral anticoagulants)	4 (31)	7 (23)	
Combination of antiplatelet and anticoagulant agents	3 (23)	7 (23)	
No antithrombotic agent	1 (8)	4 (14)	

Higher M2BPGi group, M2BPGi ≥ 0.64 C.O.I; Lower M2BPGi group, M2BPGi < 0.64 C.O.I.

C.O.I = cutoff index, M2BPGi = Mac-2 binding protein glycosylation isomer.

**Table 4:** Correlation of M2BPGi with other parameters.

Variables	Correlation coefficients (r)	*P*-value
Age at evaluation [years]	0.274	.076
Age at the Fontan procedure [years]	0.558	<.001
Duration of Fontan circulation [years]	-0.121	.441
Body mass index [kg/m^2^]	0.225	.147
Cardiac parameters		
Central venous pressure (mm Hg)	0.413	.006
Ejection fraction (%)	0.040	.801
O2 saturation (%)	-0.276	.073
Brain natriuretic peptide (pg/mL)	0.431	.004
Laboratory data		
Total bilirubin [mg/dL]	-0.062	.695
Aspartate aminotransferase [U/L]	0.321	.036
Alanine aminotransferase [U/L]	-0.019	.902
Alkaline phosphatase [U/L]	0.246	.112
Gamma-glutamyl transferase [U/L]	0.017	.912
Albumin [g/dL]	-0.562	<.001
Platelet count [x10^4^/μL]	-0.247	.110
Serological fibrosis markers		
FIB-4 index	0.492	.001
APRI	0.399	.008

Data are presented as numbers for categorical data, and medians with interquartile ranges for continuous variables.

APRI = aspartate aminotransferase to platelet ratio index, FIB-4 = fibrosis-4, M2BPGi = Mac-2 binding protein glycosylation isomer.

### 3.5. Evaluation of the correlation of M2BPGi with VAST score

**Figure 2.**
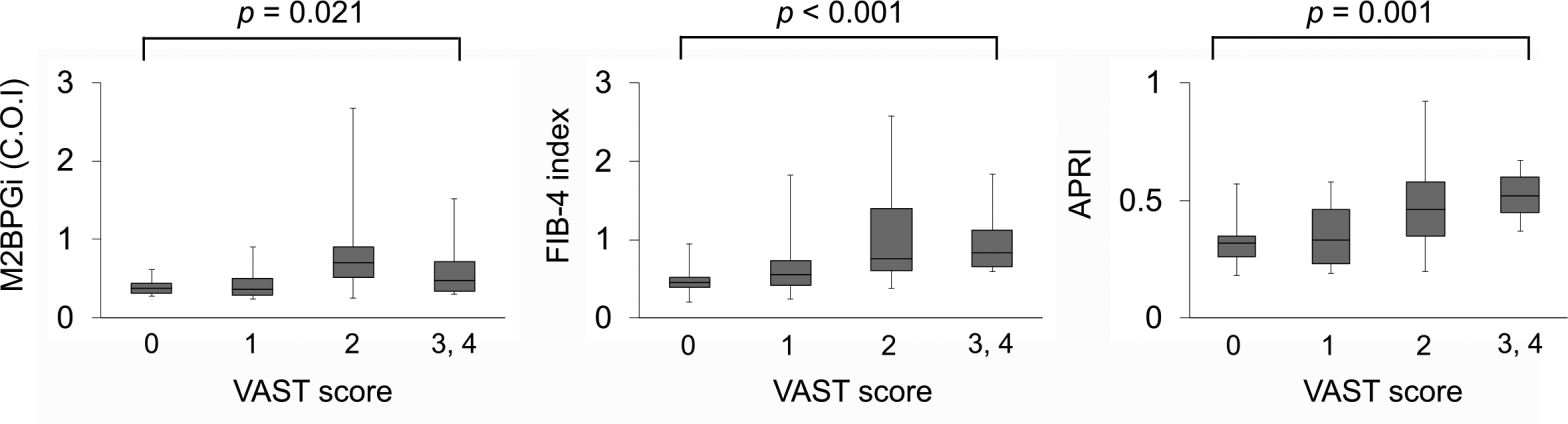
The value of M2BPGi, FIB-4 index, and APRI according to the VAST score. The values of VAST score of 3 and 4 were combined because only one case had a VAST score of 4. There were 11 cases with a VAST score of 0, 10 cases with a score of 1, 15 cases with a score of 2, and 7 cases each with scores of 3 and 4. M2BPGi, Mac-2 binding protein glycosylation isomer; C.O.I, cutoff index; FIB-4, fibrosis-4; APRI, aspartate aminotransferase to platelet ratio index.

We also evaluated the relationship between the VAST score and M2BPGi levels using the trend test. As shown in Figure 2, the Jonckheere-Terpstra test demonstrated a significant trend in increasing the value of M2BPGi with increasing VAST scores (*P* = .021). The FIB-4 index and APRI values also demonstrated a significant trend with increasing VAST scores (FIB-4 index, *P* < .001; APRI, *P* = .001). The M2BPGi levels for each VAST score were 0.38 C.O.I for zero, 0.36 C.O.I for one, 0.70 C.O.I for 2, and 0.48 C.O.I for 3 and 4, respectively.

## 4. Discussion

In the present study, we evaluated the usefulness of M2BPGi in identifying cases with advanced FALD. We demonstrated that cases with advanced FALD defined by the VAST score had elevated M2BPGi levels and that M2BPGi had a moderate ability to predict advanced FALD. Moreover, M2BPGi demonstrated a significant correlation with cardiac function indicators such as CVP and BNP, and the FIB-4 index and APRI, which have been reported to be useful in identifying FALD cases with advanced fibrosis.^[9,10]^ Furthermore, a significant trend was observed between M2BPGi levels and the VAST score. Therefore, we believe that M2BPGi is useful for identifying advanced FALD.

There is no established definition for advanced FALD. Several previous reports have used the definition of the EASL-ERN position paper.^[15]^ This position paper proposes defining advanced FALD based on the clinical signs of portal hypertension and using the VAST score.^[14,15]^ The VAST score is a scoring system based on the presence of esophagogastric varices, ascites, splenomegaly, and thrombocytopenia as clinical signs of portal hypertension, and a VAST score ≥ 2 indicates a poor prognosis in cases with FALD.^[16]^ The VAST score is often used to evaluate liver fibrosis and predict prognosis in FALD.^[20–22]^ Liver biopsy and elastography are often performed to diagnose the progression of liver fibrosis. However, liver biopsy is difficult to perform because of the risk of bleeding, and elastography has problems such as being affected by congestion and the lack of an established cutoff value in FALD. In our study, cases with advanced FALD defined by the VAST score had high FIB-4 index and APRI, suggesting the progression of liver fibrosis. Therefore, we believe that the VAST score is useful for predicting advanced FALD.

In this study, M2BPGi was significantly higher in cases with advanced FALD, and ROC analysis demonstrated that it had the equivalent ability to predict advanced FALD as FIB-4 index and APRI. M2BPGi is secreted by HSCs and promotes M2BP expression in Kupffer cells, activating HSCs and promoting liver fibrosis.^[11,12]^ HSCs also play a central role in the progression of fibrosis in congestive liver diseases.^[2]^ Therefore, we believe that M2BPGi is useful for predicting fibrosis in FALD. M2BPGi has been suggested to be useful for predicting of HCC development in viral hepatitis and MASLD.^[13]^ In this study, the usefulness in predicting HCC development in FALD could not be investigated because there were no cases of HCC. Further investigation is required into the usefulness of M2BPGi in predicting HCC development in FALD. Moreover, M2BPGi was significantly correlated with cardiac parameters such as CVP and BNP. CVP and BNP are important indicators for evaluating Fontan circulation because they closely reflect the severity of congestive heart failure.^[23]^ It is suggested that M2BPGi may not only be a fibrosis marker, but also reflect the severity of cardiac function in FALD patients. In advanced FALD, many patients die from heart failure or require heart transplantation.^[24]^ Therefore, careful monitoring of not only liver but also cardiac function is required in cases with advanced FALD. In our study, M2BPGi was more strongly correlated with cardiac function than FIB-4 index or APRI. M2BPGi may be a useful marker in advanced FALD because it can monitor both liver and cardiac function.

M2BPGi is useful for predicting liver fibrosis in various chronic liver diseases, but the cutoff value differs depending on etiology.^[25]^ The cutoff value obtained from ROC analysis in our study was lower than that reported for chronic liver disease.^[12]^ In the previous studies, the cutoff values of M2BPGi for predicting severe fibrosis were 4.0 C.O.I for HCV, 1.2 C.O.I for MASLD, and 1.2 C.O.I for hepatitis B virus.^[12,13]^ All of these values were higher than the cutoff values for identify advanced FALD obtained in this study. M2BPGi is affected not only by liver fibrosis but also by inflammation in the liver.^[26]^ However, FALD is a pathology characterized by liver fibrosis without inflammation.^[3,4,7]^ The cutoff values of FIB-4 index and APRI were also lower than those previously reported in patients with chronic liver disease.^[27]^ These fibrosis scores in the previous studies were also low in FALD.^[9,28]^ The differences in fibrosis progression between FALD and other chronic liver diseases may be contributing to the differences in serological fibrosis markers, including M2BPGi. In congestive liver disease, the worsening of congestion leads to hypoperfusion of the liver and hypoxic liver damage, leading to impaired transaminase clearance and a decrease in serum albumin levels.^[29,30]^ In our study, M2BPGi correlates with CVP, BNP, AST, albumin, and the prevalence of esophagogastric varices and ascites, suggesting that these indicators deteriorate with the progression of liver fibrosis and deterioration of cardiac function in FALD. Therefore, it is presumed that transaminase, cholestatic enzyme, and fibrosis markers are low in the early stages of FALD, but deteriorate due to the progression of fibrosis caused by sustained liver congestion and ischemia. However, the pathogenesis of liver fibrosis in FALD is complex and requires further investigation.

In this study, the age at Fontan procedure was significantly older in cases with advanced FALD, and demonstrated a positive correlation with M2BPGi levels. Currently, the optimal age for Fontan procedure is considered to be around 2–3 years old.^[31]^ The median age at Fontan procedure in cases with non-advanced FALD was 2.6 years in our study. Therefore, the optimal age for Fontan procedure is considered appropriate. It has been reported that the progression of liver fibrosis in FALD correlates with the duration of Fontan circulation,^[9,10]^ but significant difference was not observed in our study. The average time until a diagnosis of liver cirrhosis after the Fontan procedure is reported to be 23.4 years,^[32]^ but the cases after more than 20 years from the Fontan procedure were only seven (16%) in our study. Therefore, long-term observation is required because the long Fontan circulation may be related to advanced FALD.

This study has some limitations. First, this was a retrospective and a single-center study with a limited number of patients. Moreover, selection bias may exist because this study included only patients who consulted a hepatologist. It is difficult to investigate a large number of FALD cases at a single institution. The results need to be validated in multiple facilities. Second, there were no cases in which liver biopsy was performed. Liver biopsy is the gold standard for evaluating liver fibrosis, but it is not easy to perform in FALD because of the risk of bleeding. Third, there were no cases developing HCC in this study. In managing FALD, the most important concern is the concomitant development of HCC. Therefore, further investigation is required to determine whether advanced FALD defined by the VAST score are at high risk of HCC. Fourth, this study evaluated the parameters used at the diagnosis of FALD, but FALD is a condition that worsens insidiously over time after the Fontan procedure. Therefore, the temporal changes in fibrosis markers are also an important consideration.

## 5. Conclusion

This study demonstrated that M2BPGi may be useful in identifying advanced FALD. Moreover, M2BPGi also correlates with cardiac function, suggesting that it may not simply be a fibrosis marker in FALD. We believe that M2BPGi is a useful serological marker in FALD.

## Author contributions

**Conceptualization:** Kensuke Kitsugi, Kazuhito Kawata.

**Data curation:** Kensuke Kitsugi.

**Formal analysis:** Kensuke Kitsugi.

**Investigation:** Kensuke Kitsugi.

**Methodology:** Kensuke Kitsugi.

**Project administration:** Kensuke Kitsugi.

**Supervision:** Kazuhito Kawata.

**Visualization:** Kensuke Kitsugi.

**Writing – original draft:** Kensuke Kitsugi.

**Writing – review & editing:** Yoshisuke Hosoda, Go Murohisa, Yashiro Yoshizawa, Masaharu Kimata, Yosuke Kobayashi, Shuhei Unno, Yosuke Yamada, Toshihiro Takayanagi, Kazuhito Kawata.
